# The importance of accurate road data for spatial applications in public health: customizing a road network

**DOI:** 10.1186/1476-072X-8-24

**Published:** 2009-05-01

**Authors:** Brian G Frizzelle, Kelly R Evenson, Daniel A Rodriguez, Barbara A Laraia

**Affiliations:** 1 Carolina Population Center, CB# 8120, University of North Carolina-Chapel Hill, Chapel Hill, North Carolina, USA; 2 Department of Epidemiology, Gillings School of Global Public Health, University of North Carolina-Chapel Hill, Chapel Hill, North Carolina, USA; 3 Department of City and Regional Planning, University of North Carolina-Chapel Hill, Chapel Hill, North Carolina, USA; 4 Department of Medicine, Division of Prevention Sciences, Center for Health and Community, University of California, San Francisco, California, USA

## Abstract

**Background:**

Health researchers have increasingly adopted the use of geographic information systems (GIS) for analyzing environments in which people live and how those environments affect health. One aspect of this research that is often overlooked is the quality and detail of the road data and whether or not it is appropriate for the scale of analysis. Many readily available road datasets, both public domain and commercial, contain positional errors or generalizations that may not be compatible with highly accurate geospatial locations. This study examined the accuracy, completeness, and currency of four readily available public and commercial sources for road data (North Carolina Department of Transportation, StreetMap Pro, TIGER/Line 2000, TIGER/Line 2007) relative to a custom road dataset which we developed and used for comparison.

**Methods and Results:**

A custom road network dataset was developed to examine associations between health behaviors and the environment among pregnant and postpartum women living in central North Carolina in the United States. Three analytical measures were developed to assess the comparative accuracy and utility of four publicly and commercially available road datasets and the custom dataset in relation to participants' residential locations over three time periods. The exclusion of road segments and positional errors in the four comparison road datasets resulted in between 5.9% and 64.4% of respondents lying farther than 15.24 meters from their nearest road, the distance of the threshold set by the project to facilitate spatial analysis. Agreement, using a Pearson's correlation coefficient, between the customized road dataset and the four comparison road datasets ranged from 0.01 to 0.82.

**Conclusion:**

This study demonstrates the importance of examining available road datasets and assessing their completeness, accuracy, and currency for their particular study area. This paper serves as an example for assessing the feasibility of readily available commercial or public road datasets, and outlines the steps by which an improved custom dataset for a study area can be developed.

## Background

Over the last two decades, the public health field has increasingly adopted the use of Geographic Information Systems (GIS) for analyzing environments in which people live and how those environments affect health. One subset of this research focuses on the impacts of road networks, examining accessibility to health care along those networks [1-3]. Another area of research has examined the geographic location of road networks relative to other locations of interest, such as places of residence, schools, or other community facilities, and how such proximity affects health outcomes. Specifically, this research has used road networks in GIS to examine exposures such as traffic [4], air pollution [5-8], and degree of urbanicity [9] and to study health outcomes such as physical activity [9-13], respiratory, pulmonary and cardiovascular morbidity and mortality [14-26], pre-term birth [27,28], and childhood cancers [29-32].

**Figure 1:**
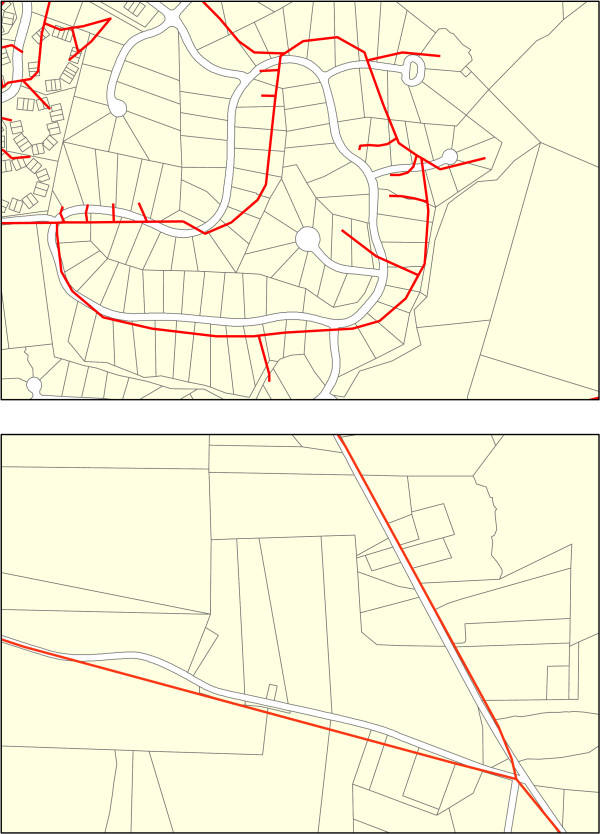
**Examples of Positional Error and Generalization, with Roads overlaid on Tax Parcels**. Figure 1a is an example of positional error, while Figure 1b is an example of generalization. Roads (in red) are overlaid on tax parcels (road locations in white).

One aspect of this research that is often overlooked is the quality and detail of the road data and whether or not it is appropriate for the scale of analysis. Many readily available road datasets, both public domain and commercial, contain positional errors or generalizations that may not be compatible with highly accurate geospatial locations. Positional errors occur when the coordinates of the vertices that define the shape of a given road feature are incorrect, indicating that the shape and position of the road segment differs from reality (Figure 1a). Generalizations occur when road features do not contain enough vertices to accurately delineate the curvilinear nature of the feature (Figure 1b). Additionally, some datasets were created for non-analytical purposes, and their use in detailed analysis is inappropriate. Most GIS datasets have accompanying metadata which describes the purpose of the dataset and the scale of the data or the positional accuracy (i.e., how closely the coordinates match reality) of its features, and this can be used to determine its suitability. For example, prior to the Master Address File/Topologically Integrated Geographic Encoding and Referencing Accuracy Improvement Project (MAF/TIGER^® ^AIP) [33], the US Census Bureau's TIGER/Line files contained large positional errors [34-36]. Though many public health studies used TIGER/Line data prior to this improvement in positional accuracy [e.g. [37-39]], few alternatives existed until early in this decade. In addition, even with improvements, most of these datasets are often not as current as needed, and contain both omissions of existing roads and commission of non-road features (e.g., driveways, power lines, railroads, rivers). Unfortunately, it is difficult to find concise quantitative information on the amount of inclusion – or conversely exclusion – of road features in any given road dataset [40].

Several studies have assessed the positional accuracy or error of digital line data. Efforts to examine accuracy are limited in their applicability because they either require a large expenditure of effort to calculate accuracy, or they require a consistency and comparability in data attribution that may not be present among most road datasets. These studies include the development of models for calculating positional error of line segments [41,42], calculating distances between road segments matched by road name [36], assessing error via the distances between intersections in the data and reference points collected at road intersections with global positioning system (GPS) [43] or from aerial photography [44], and comparing road density to a very detailed road network built manually for such a purpose [40]. In general, there does not appear to be a proven method of effectively assessing positional accuracy for a wide variety of digital line features such as roads. Since positional error distributions are typically not normally distributed, and vary greatly depending on the type of feature, the methods for assessing positional accuracy will likely depend on the source of the spatial data being analyzed and the method by which is was created [44].

This study examines the accuracy, completeness, and currency of four readily available public and commercial sources for road data relative to a custom road dataset that we used for comparison. The customized road dataset was built for the purpose of analyzing the association of health behaviors with the built environment among women living in central North Carolina in the United States from pregnancy to postpartum [45]. The development of a road dataset tailored to the specific needs of this project was a result of the observation that other available public and commercial road datasets were insufficient in terms of their positional accuracy and completeness of features. Many of these public and commercial datasets are readily available and are used for spatial analysis in public health research. This paper serves as a guide for assessing the feasibility of readily available commercial or public road datasets, explains their limitations, discusses the circumstances under which the development of an improved custom road dataset would be beneficial, and outlines the steps to develop such a dataset.

## Methods

### Data Requirements

The unique locations of 2,444 residences, representing homes for 1,491 participants over three time periods, were identified via GPS or address geocoding. Each residential location was subsequently moved manually, if necessary, to lie within 50 feet (15.24 meters) of the road segment on which they lived, falling on or near the driveway. We required that all participant home locations be within 50 feet (15.24 meters) of their road segment to facilitate GIS analyses. Neighborhoods were participant-specified, defined as the area covered by travelling up to 1 mile (1.6 kilometers) on the road network.

Accuracy, completeness, and currency are critical elements in this study. Accuracy is important because we investigated the nature of relatively small neighborhoods. The actual size of neighborhoods depended on the road connectivity around each participant's home, ranging from 0.05 square miles (0.13 km^2^) and 3.14 square miles (8.14 km^2^). Completeness is important because the study area of Alamance, Chatham, Durham, and Orange counties is rapidly growing and includes land uses ranging from high-density urban (e.g., the city of Durham) to low-density rural. The development of single-family home subdivisions is prevalent throughout the four counties and home construction has been widespread and consistent for more than a decade. Therefore, the road network is very dynamic, with new roads being built often, and that requires that road datasets be updated on a regular basis. Currency is important because a road network that is not maintained and updated regularly will not capture all new roads as they are built. In such a dynamically changing area such as our study area, road data can quickly become outdated.

### Road Data

#### Publicly and Commercially Available Road Datasets

Three road network datasets were considered for use in the project during the database development phase in 2005. The three datasets were the North Carolina Department of Transportation (NC DOT) road data, the ESRI StreetMap Pro road data, and the US Census Bureau's TIGER/Line roads from the year 2000. Table 1 contains general characteristics of these three datasets plus the more current TIGER/Line 2007 data.

**Table 1:** Summary information on the four publicly and commercially available road datasets

Road Data	Spatial Extent	Currency	Availability	Source
NC DOT	North Carolina	2005	Public	North Carolina Dept. of Transportation

StreetMap Pro	USA	2003	Commercial	ESRI

TIGER/Line 2000	USA	2000	Public	U.S. Census Bureau

TIGER/Line 2007	USA	2007	Public	U.S. Census Bureau

The NC DOT road dataset maintained by the state of North Carolina covers all 100 counties. It is a public domain dataset, available for free download from the NC DOT website [46]. NC DOT data is available both county-by-county and as a comprehensive statewide layer. The metadata accompanying the dataset states that its purpose is to "assist governmental agencies and others in making resource management decisions through the use of a Geographic Information System... [via] location analysis." [47] We selected the most recent version at that time, which was current to 2005.

The US Census TIGER/Line road data are a subset of the TIGER/Line line features dataset maintained by the US Census Bureau in Washington, D.C. All TIGER/Line data have a very generic purpose, to be used for "geographic applications." [48] As with the NC DOT data, it is a free public domain, and must be downloaded county by county. TIGER/Line files contain a multitude of geographic features for the entire United States, but it is possible to subset out particular features for specific needs, such as roads in this case. The TIGER/Line datasets historically contain many errors of spatial accuracy and completeness, especially in rural areas [49]. However in 2004, as part of the MAF/TIGER AIP, the First Addition of the TIGER/Line data began incorporating "realigned street feature coordinates" from counties or statistically equivalent entities [33]. We chose the 2000 TIGER/Line data because it was readily available at the time of the study.

The ESRI StreetMap Pro data (now called Streetmap North America) are a commercially maintained dataset, available for purchase on a licensed basis from ESRI. StreetMap Pro is based on Tele Atlas data and covers the entire United States and Canada. The stated purpose of this dataset is to be used for "display, routing and geocoding." [50] The StreetMap Pro road features are current as of 2003.

All of these datasets were considered insufficient to suit the spatial needs of the project. We performed a visual investigation, such as described by Hawbaker and Radeloff [40], by overlaying participant locations on each of the road networks, and also comparing roads to aerial photographs and county tax parcel data. We determined that all three datasets were missing a large number of local roads, both rural and suburban, on which participants lived. The positional accuracy of the StreetMap data was poor in rural areas, while the TIGER/Line 2000 positional accuracy was poor in all portions of the study area through visual inspection. The NC DOT and StreetMap Pro datasets included many non-road features in rural areas, and the TIGER/Line 2000 features were highly generalized and not reflective of the existing road network.

In this analysis, we also considered the 2007 TIGER/Line road data as an additional comparison. However, these road data were not available at the time we were evaluating datasets. These data are a result of the US Census Bureau's plan to use locally derived feature data to increase positional accuracy to meet their stated goals that 95% of the features be within 7.6 meters of their true locations [51]. Our intent in adding this dataset to the analysis is to provide readers with additional information when deciding which dataset is most appropriate for their work.

#### Customized Road Network

Since the NC DOT, TIGER/Line, and StreetMap Pro datasets did not meet our criteria of completeness or accuracy when we conducted the study, county-level road centerline datasets were obtained for the four-county study area. These datasets are developed and maintained by local county departments and designed to be compatible with the county-maintained tax parcel datasets. Visual comparison of the county centerline files with the larger, more expansive datasets revealed that the county data were superior to the others in both positional accuracy and currency of road features. We therefore chose to combine the various county datasets – from Alamance, Chatham, Durham, and Orange counties – into one layer covering the full study area.

The Alamance road data were developed by the Alamance County GIS Department and made available for download from their website. They were current as of 2004, and the initial review of the data showed some geographic errors (e.g. inclusion of non-road features) and quite a few attribute errors (e.g. no names on many rural roads). According to the metadata accompanying the dataset, the positional accuracy of the dataset conformed to the Technical Specifications for Base, Cadastral, and Digital Mapping document produced by the NC Land Records Management Program [52].

The Chatham County road data were produced by the Chatham County GIS Department, the Durham County road data were produced by the Durham County GIS Department, and the Orange County road data were produced by the Orange County GIS Department. All three of these datasets were current as of 2005. None of the datasets were accompanied by metadata, so we had no information on the scale of usage or on the assessed positional accuracy of the features. However, simple overlays with tax parcels and aerial photographs assured us that the accuracy was more than sufficient for our needs.

**Figure 2:**
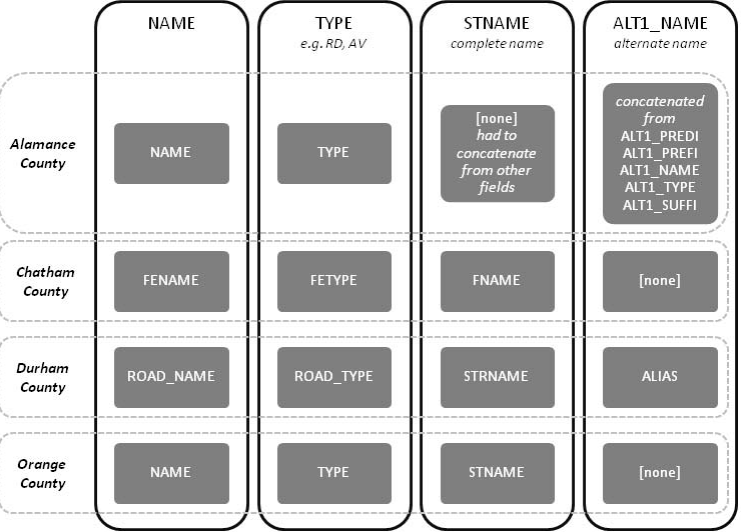
**Example of Attribute Field Normalization**. The columns represent attribute fields that we desired, and the gray rectangles within show the names as they appeared in the source datasets.

One problem associated with the merging of disparate datasets is that the attribute data is often mismatched and is rarely comparable. This was the case with the four county datasets. There are no international, national, or even statewide regulations on the creation and maintenance of spatial datasets, especially regarding attribute fields. Therefore, we selected the attribute fields that would be most beneficial to the project and identified the field or fields in each of the four datasets that best matched our desired fields. Figure 2 shows an example of this. Those fields were then normalized across all four datasets and the remaining fields removed.

The four datasets were edge-matched and appended together to create a study area-wide road network dataset. Edge-matching is a spatial adjustment method that aligns features along the edge of adjacent datasets so that they can be merged together. Appending is a spatial editing method that merges multiple datasets of the same data type together into one larger dataset. The baseline dataset was overlaid on tax parcels and aerial photography to identify and delete non-road features that were erroneously included in the data. To make the dataset topologically sound, pseudo-nodes and overlapping line features were removed and intersections were created where two roads crossed but did not intersect. Subdivisions, apartment complexes, trailer parks, or other legitimate networks of roads on which people live were added when discovered to be missing from the data. Road features were modified and reshaped to more accurately fit reality when incorrect. This included updating roads that had been rerouted and widened from two-lane to four-lane-divided, lengthening roads that did not extend to their full length, and shortening roads that extended too far. Updates were performed through GIS overlays with aerial photographs and tax parcels, and by obtaining road locations in the field with differentially corrected GPS data collected with mapping grade Trimble GPS receivers.

### Spatial and Statistical Analyses

The goal of our analysis was to quantitatively compare the four commercially and publicly available road network datasets to the custom road network data. It should be noted that none of these methods are designed to assess the error of individual road segments. While there are studies that have done this [36,40-44], the time investment required for such an endeavour was considered to too large to justify the minimal gain in understanding the nature of the errors. Therefore, the methods that were chosen provide sufficient information on the spatial distribution of errors and are easier to implement.

We used three measures, described below, to comparatively assess the accuracy and completeness of the various publicly and commercially available datasets. The first two were localized, neighborhood-based methods that used a Euclidean buffer with a radius of 402 meters (0.25 miles) around each participant location to calculate local metrics that informed us of dataset comparability as it pertains to our participants. The third method was a more comprehensive approach that compared the datasets in their entirety to one another. We used the customized road network as the comparison dataset.

The first measure was distance from each participant's home to the nearest road, calculated for all road datasets. This Distance to Nearest Road measure allowed us to easily identify how many participants would not be located on their road if any of the other datasets were used. Recall that we hand corrected our participant locations to within 15.24 meters (50 feet) of their road feature in the customized road network. Therefore, we can say with confidence that any participant located farther than 15.24 meters from the nearest road might have been excluded from some of the network analyses if they were performed with a different road dataset. However, the alternative would have been to increase the search threshold for the network analysis, which would likely have resulted in some locations being assigned to the wrong roads. We calculated the percentage of participants whose nearest road was farther than that threshold distance, but compensating for slight differences in positional accuracy which may not have significantly impacted our research, we also calculated those percentages using thresholds of 50% and 100% larger than our 15.24 meters.

Total Road Length was our second measure. It was calculated within the Euclidean buffer around each of the participant locations. This measure was used in lieu of a density measure as all buffers were the same size and therefore the relationships were identical in the two measures. Comparatively, a significant deviation in total length between a readily available dataset and the customized data indicated that the available data omitted features, included non-road features, or had positional errors in the features that could be random or systematic. Thresholds values (deltas) were set for identifying those neighborhoods with divergent road networks.

**Figure 3:**
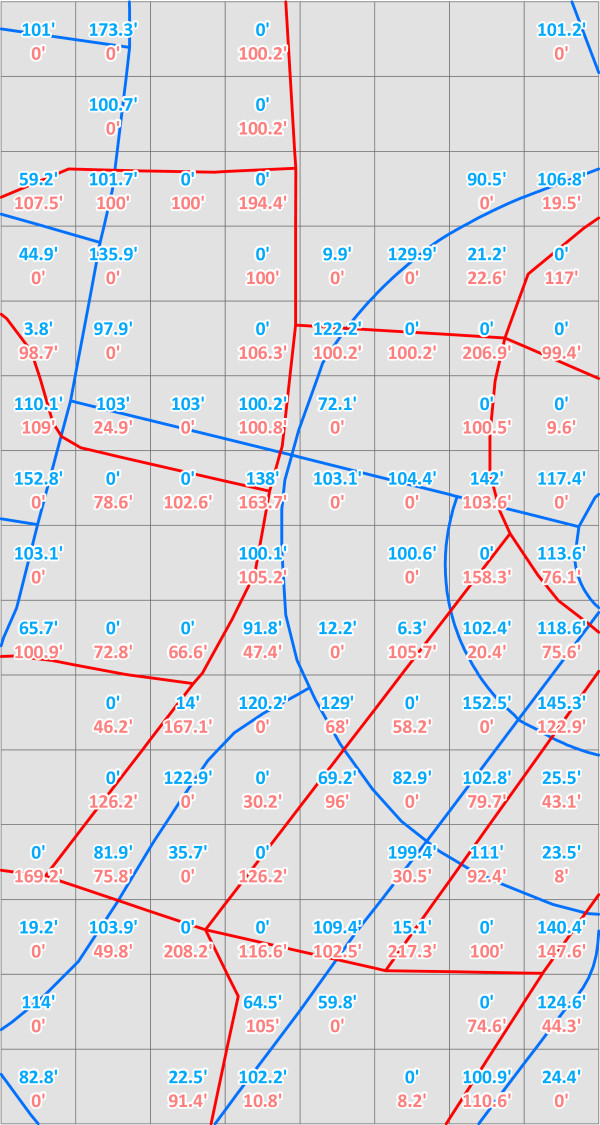
**Summation of Road Lengths within 100' Cells for Two Datasets in the Same Area**. The blue roads are the custom dataset and the red roads are the TIGER/Line 2000 dataset. The labels give the summed length of each dataset within the cell, with the blue labels for the custom roads and the red labels for the TIGER/Line 2000 roads.

Cell Length Summation was the third measure, which compared the positional accuracy and inclusion/exclusion of features in each of the readily available datasets to that of the customized road network. This measure summed the length of all road segments inside 100-foot vector cells, which were used to mimic a raster dataset. Figure 3 shows an example of this by comparing the customized road network and the TIGER/Line 2000 road network for the exact same area, and the differences in summed lengths (values in the cells) can be compared between the two. Vector cells were used instead of raster data because there is no proven tool available for summing vector line lengths within raster cells. There is a tool, however, within the Hawth's Analysis Tools for ArcGIS toolkit [53] that sums line lengths by polygon. Therefore, a regular lattice array of square polygons, 100 feet on a side, was generated over the study area, creating a dataset containing more than 6.73 million cells. The "Sum Line Lengths in Polygon" tool [53] was used to calculate the total length of road segments for each of the five datasets within each cell. Agreement between cell length summation calculated with the customized road data and each of the four other road datasets was explored using concordance coefficients and Pearson correlation coefficients [54,55]. The concordance correlation coefficient combines measures of both precision and accuracy to determine how far the observed data deviate from perfect agreement, thus correcting for chance agreement. The calculated coefficient increases in value as a function of the accuracy and precision of the data. As a rough guide, we used the ratings suggested by Landis and Koch for agreement [56]: 0–0.2 poor, 0.2–0.4 fair, 00.4–0.6 moderate, 0.6–0.8 substantial, and 0.8–1.0 almost perfect.

The spatial distribution of the errors was then investigated using the Cell Length Summation results. In these analyses, the dependent variable was each cell's sum of the road length for the custom dataset, and the independent variables included the sum of each comparison dataset's roads. Only those cells containing roads from at least one of the five datasets were used, which reduced the sample size from the full 6.73 million to 729,597 observations. From the four linear regression models, the studentized residuals were calculated and assigned to each cell. The residuals summarize the standardized error in each cell between the custom dataset and the comparison dataset.

## Results

The Distance to Nearest Road measure indicated that each of the four comparison road datasets excluded enough road features to cause potential problems with network analyses if used with the participant locations. Table 2 shows that the mean distance from a participant's home to the nearest road exceeded the maximum allowed distance of 15.24 meters (50 feet) in three of the four comparison datasets. In the fourth comparison dataset (NC DOT), the mean distance was slightly below the maximum allowed distance, yet it still had one participant whose closest road was 395 meters away.

**Table 2:** Mean, standard deviation, and maximum distance from the participant's home to the nearest road using five different road datasets (n = 2,444, participant locations)

Road Data	Mean in m	Standard. Deviation in m	Maximum in m
Custom Data	7.89	3.34	15.17

NC DOT	13.10	28.92	395.06

StreetMap Pro	22.68	49.01	444.43

TIGER/Line 2000	50.04	73.08	600.32

TIGER/Line 2007	15.54	37.82	777.56

Further analysis showed that the StreetMap Pro and TIGER/Line 2000 datasets contained an unacceptable percentage of nearest road segments that were beyond the 15.24 meter threshold (Table 3). In all four comparison datasets, the majority of those participants who were greater than 15.24 m from a road were more than double the threshold distance (30.48 m) from that road. This indicates the number and percentage of participants that might have had additional error introduced to their measures if we had used each of these road datasets.

**Table 3:** Threshold analysis from Distance to Nearest Road measure that assesses the distance from the participant's home to the nearest road (n = 2,444 participant locations)

Road Data	Observations > 15.24 m (%)	Observations 15.24 m – 22.86 m (%)	Observations 22.86 m – 30.48 m (%)	Observations > 30.48 m (%)
Custom Data	0 (0)	0 (0)	0 (0)	0 (0)

NC DOT	145 (5.9)	30 (1.2)	7 (0.3)	108 (4.4)

StreetMap Pro	463 (18.9)	139 (5.7)	34 (1.4)	290 (11.9)

TIGER/Line 2000	1,548 (63.3)	320 (13.1)	208 (8.5)	1,020 (41.7)

TIGER/Line 2007	234 (9.6)	46 (1.9)	30 (1.2)	158 (6.5)

**Figure 4:**
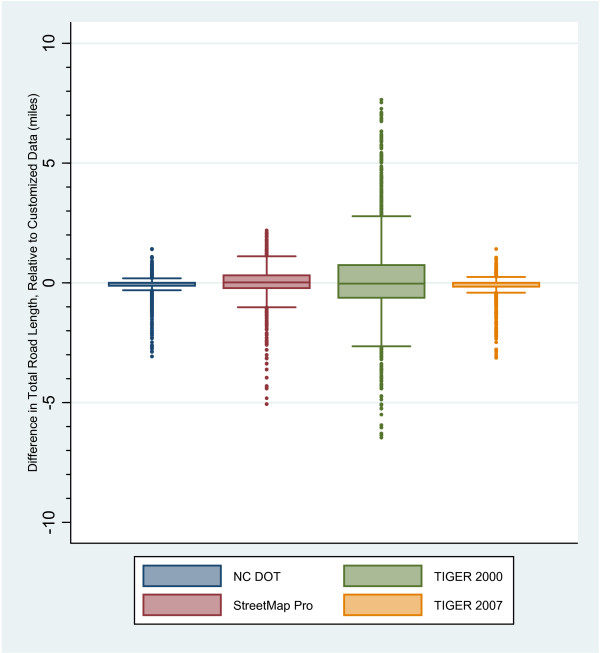
**Box Plot Comparison of Road Length Differences**.

After calculating Total Road Length, the difference between each comparison dataset and the customized dataset was calculated in each participant neighborhood. The difference for each road dataset was then divided by the length of the customized road segments to convert the difference to a percentage of the customized road total length. Nearly 80% of the neighborhoods had total road lengths from the NC DOT and TIGER/Line 2007 datasets that were within 10% of the customized total road length, whereas the Streetmap Pro data lengths were within 10% in 57% of the neighborhoods and the TIGER/Line 2000 roads were within 10% in 32% of the neighborhoods. In addition, the TIGER/Line 2000 data deviated by more than 100% of the customized total road length in almost 12% of the neighborhoods. Comparing the distribution of these differences side by side (Figure 4), both the NC DOT and TIGER/Line 2007 data had a narrow distribution around the 0 value, which indicated high comparability with the customized road data. While all four datasets contained a large number of outliers, it was the larger distributions of differences in the StreetMap Pro and TIGER/Line 2000 datasets that indicated that they compared less favorably in this analysis.

We measured agreement between the Cell Length Summation values across the 729,597 vector cells that contained at least one of the five road datasets (Table 4). We found that agreement between the customized road data and the NC DOT road data was the highest, with near perfect agreement. TIGER/Line 2007 data had substantial agreement with the customized road data, the StreetMap Pro data had moderate agreement, and agreement with TIGER/Line 2000 data was poor.

**Table 4:** Total road length agreement between customized and other road datasets in vector cells containing at least one road dataset (n = 729,597 cells)

	Concordance coefficient (p-value)	Pearson Correlation Coefficient (p-value)
NC DOT	0.82 (< 0.001)	0.83 (< 0.001)

StreetMap Pro	0.60 (< 0.001)	0.60 (< 0.001)

TIGER/Line 2000	0.01 (< 0.001)	0.01 (< 0.001)

TIGER/Line 2007	0.79 (< 0.001)	0.79 (< 0.001)

To examine geographically the areas with low agreement from the customized data, we used the residuals from univariate regressions of the cell length sum of the customized road data as dependent variable and each of the comparison datasets as independent variables. We explored additional regression analyses using 2000 US Census block group mean-centered socio-economic and urbanization variables to examine whether they explained the residuals calculated (results not shown), but none of the independent variables had meaningful relationships with the errors.

**Figure 5:**
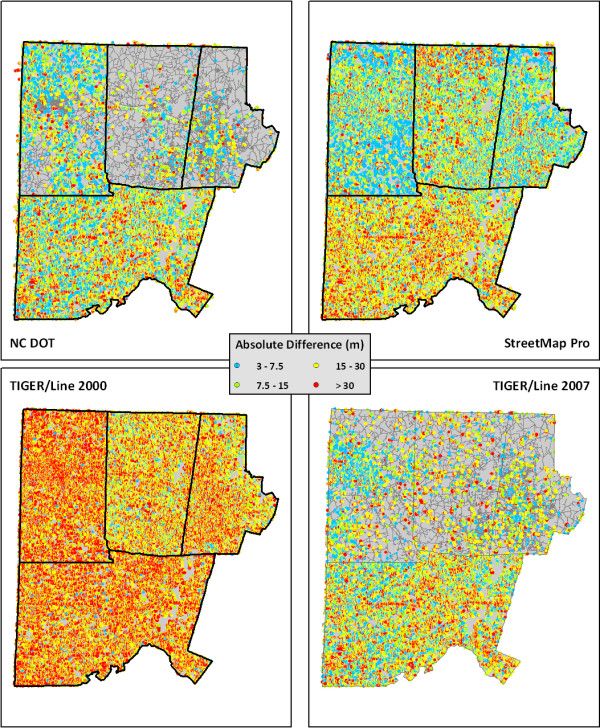
**Absolute Difference (meters) In Cell Length Summation Values Between Custom Road Dataset and Comparison Road Datasets**. The map is zoomed in to the central part of the study area. Any areas where colored dots do not appear over the roads have less than a 3 meter difference between the two datasets.

Finally, we mapped the absolute cell length sum differences for each comparison pair to further examine the geographic distribution of error (Figure 5). Only cells with differences greater than 3 meters are shown as colored dots, so areas where the roads can be seen clearly have very little error. The figure confirms that there is less error across the study area for the NC DOT and TIGER/Line 2007 datasets, whereas the TIGER/Line 2000 dataset has the greatest error throughout the entire study area. The magnitude of error is also less for the NC DOT and TIGER/Line 2007.

## Discussion

There are a number of benefits to using prepackaged comprehensive road data in research studies. These road datasets often cover very large areas, whether at the state or national level, and the associated attribute information is often clear and consistent. Often they are topologically sound, meaning that all roads are connected at intersections, creating a complete network that facilitates GIS network analysis. However, there are some important limitations as well. We found that these prepackaged datasets are often out of date, are missing new roads, and contain positional errors which can cause erroneous analysis results. By comparing these datasets to a custom designed road network dataset that was both current and accurate, we discovered that errors of positional accuracy, omission and commission occur more often and with greater magnitude in rural areas and in suburban areas experiencing rapid change and growth.

So how does one go about anticipating problems with available road datasets and then determining whether or not to create a custom road dataset? There are a several points to consider. First, determine the purpose of the original datasets and the reported scale and positional accuracy. This information should be provided in the accompanying metadata, although not all spatial datasets have metadata files. Small-scale datasets, meaning those that cover very large areas, tend to have generalized features and larger positional error in their features. Conversely, large-scale datasets, those that cover small areas, tend to have more detailed features and less positional error [57]. But scale is a tricky concept for digital data, because small-scale data can be subset for a small area and still contain the original errors. For example, if a road dataset for the United States has a scale of 1:1,000,000, it will have very generalized and inaccurate features and will likely be missing many local roads. But if someone were to subset those data for the city of Chicago, it now covers a small area but it still contains the same errors and is still subject to the small-scale limitations. Positional accuracy, often referred to as horizontal accuracy in metadata, is a simpler concept to interpret. A reported horizontal error of 10 meters is an average error for a dataset, but it can be assumed it is consistent throughout and is not affected by a change in the spatial extent of the data. And in general, it is always important that all datasets, road or otherwise, have similar scales or positional accuracy.

Second, the currency of the dataset can be key. This is also reported in the metadata, and is usually called Time Period of Content or something similar. If a study area is experiencing or has experienced significant change between the date of the dataset and the current time, then it is likely that the data will not contain all of the roads needed. However, areas that do not experience much change in the road network, such as central cities, can better handle older road datasets than newer areas undergoing rapid changes. Knowledge of the study area and the nature of its growth is a key in assessing potential problems.

In any situation where the analysis focuses on locations of interest (e.g. participants, clinics, recreational facilities, etc.) with respect to a road network dataset, it is important that the source of the road network data match the means by which the locations were gathered. For example, if locations of a feature or a participant were collected with GPS receivers, then it is important to have highly accurate road centerlines, preferably those from local municipal or county departments. However, if locations were geocoded from address data in a GIS, then the most appropriate road network dataset to use with those locations would be the same road data used in the geocoding process. Even if geocoded results come from the same road data source, geocoding inaccuracies can still introduce substantial biases [58-62].

The two overriding concerns with the datasets we investigated were the level of positional accuracy and the completeness of the data. It is generally understood that most large road datasets substantially underestimate road density because they tend to omit minor roads and they are not updated regularly, missing new road development [40]. In addition, depending on the underlying source data from which the road features are based, the overall positional accuracy of the features may be less than appropriate for the study. At the time we were developing the spatial database and evaluating road datasets, all three available datasets (StreetMap Pro, NC DOT, and TIGER/Line 2000) were determined to be inadequate for the study's needs due to missing roads in many neighborhoods in which the study participants lived. In addition, the TIGER/Line data exhibited poor positional accuracy and highly generalized road features.

Based on the results of the analyses and on visual comparisons, it appears that the NC DOT and TIGER/Line 2007 data appeared to be sufficiently similar to the project dataset that they would be acceptable substitutes if no other alternative existed. However, it is still important to note that both of these datasets are still lacking in temporal currency and inclusion of new roads, and therefore the effort to create a customized road dataset that was both current and complete was the best approach. There is no single numeric measure that effectively captures the fact that many neighborhood roads on which are project participants live do not exist in one or both of these datasets, although the neighborhood-based measures do get at this problem from several different angles.

## Conclusion

There are some distinct circumstances in which the custom development of a road network data development can be considered both feasible and desirable. First, the study area must be sufficiently small. Our study area covered 4,773 square kilometers and has 11,500 kilometers of roads. This approach is not feasible for a statewide, regional, or nationwide study. Second, local knowledge of roads is a clear asset for understanding the nature of change and growth and because the project team will be able to easily and inexpensively conduct "ground truthing" or field visits with GPS receivers to collect or validate new roads or possibly erroneous road features. Third, a rural or suburban area that is experiencing moderate to rapid growth is a prime candidate for this approach. Areas such as these quickly fall out of date in road datasets. But it is ultimately up to the investigators and the project team to determine if the study area lends itself to the creation of a customized road dataset.

Having a customized road dataset provides a number of benefits. The data are more current and complete, so if the work is comprehensive, there will not be any areas within the study area where roads are not represented. There also will not be any non-road features (e.g., driveways, power lines, railroads, rivers) in the dataset. This is very important, because the inclusion of non-road features can provide alternative non-existent routes in network analysis that will skew the results and add unexpected error. However, the significant downside is the sheer amount of time and expense required to complete it.

This study demonstrates the importance of examining the available prepackaged comprehensive road datasets and assessing their completeness, accuracy, and currency for their particular study area. If enough critical roads in the study area are missing or inaccurate, then that makes a strong case for following our methodology in creating a new road dataset using data from municipalities and/or counties for the analysis.

## Competing interests

The authors declare that they have no competing interests.

## Authors' contributions

BF conducted the statistical analyses and drafted the manuscript. KE assisted with writing some sections of the manuscript. DR provided important guidance on several statistical analyses. KE, DR and BL provided critical feedback on the manuscript drafts. All authors helped conceive the study, participated in the design of the analysis, and both read and approved the final manuscript.
